# Impact of SGLT-2 inhibitors on modifiable cardiovascular risk factors in Romanian patients with type 2 diabetes mellitus

**DOI:** 10.1186/s13098-024-01326-8

**Published:** 2024-04-16

**Authors:** Adriana Gherbon, Mirela Frandes, Darius Dîrpeş, Romulus Timar, Bogdan Timar

**Affiliations:** 1 Department VII Internal Medicine - Diabetes, Nutrition, Metabolic Diseases and Systemic Rheumatology, “Victor Babes” University of Medicine and Pharmacy, Timisoara, Romania; 2 Centre of Molecular Research in Nephrology and Vascular Disease, “Victor Babes” University of Medicine and Pharmacy, Timisoara, Romania; 3 Diabetes, Nutrition, and Metabolic Diseases, “Pius Brinzeu” Emergency Hospital, Timisoara, Romania; 4 Department of Functional Sciences - Biostatistics and Medical Informatics, “Victor Babes” University of Medicine and Pharmacy, 2 Eftimie Murgu Sq., 300041 Timisoara, Romania

**Keywords:** SGLT-2 inhibitors, Cardiovascular risk factors, Diabetes mellitus

## Abstract

**Background:**

Modifiable cardiovascular risk factors are high blood pressure, smoking, diabetes, sedentary lifestyle, obesity, and hypercholesterolemia.

**Aim:**

To investigate the impact of sodium-glucose 2 co-transporter inhibitors (SGLT-2i) on modifiable cardiovascular risk factors in Romanian patients diagnosed with type 2 diabetes mellitus (T2DM).

**Method:**

A retrospective study was conducted on 200 Romanian patients with T2DM who were being treated with SGLT-2i, either Dapagliflozin or Empagliflozin. Collected data included demographic characteristics, such as weight, body mass index (BMI), fasting blood glucose (FBG), creatinine, glycated hemoglobin (HbA1c), abdominal circumference (AC), urine albumin-to‐creatinine ratio (UACR), systolic blood pressure (SBP), diastolic blood pressure (DBP), C-reactive protein (CRP) and N-terminal pro b-type natriuretic peptide (NT-proBNP). The patients were observed for one year after being treated with SGLT-2i.

**Results:**

The mean value of FBG decreased from 180.00 mg% (IQR: 154.50–207.00) to 130.00 mg% (IQR: 117.50–150.00) (*p* < 0.001), and the mean of HbA1c values decreased from 8.40% (IQR: 7.98-9.15%) to 7.30% (IQR: 6.90-7.95%) (*p* < 0.001). We also obtained significant positive effects on body weight, i.e., the weight decreased from 90.50 kg (82.00-106.50) to 89.00 kg (77.50–100.00) (*p* = 0.018), BMI from 32.87 kg/m^2^ (29.24–36.45) to 31.00 kg/m^2^ (27.74–34.71) (*p* < 0.001) and AC from 107.05 (± 16.39) to 102.50 (± 15.11) (*p* = 0.042). The UACR decreased from 23.98 mg/g (19.76–36.85) to 19.39 mg/g (1.30-24.29) (*p* < 0.001). Initially, the median value for SBP was 140.00mmgHg (130.00-160.00), and for DBP was 80.00 mmgHg (72.00–90.00), and one year after treatment, the medium value was 120.00 mmgHg (115.50–130.00) for SBP (*p* < 0.001), and 72.00 mmgHg (70.00–78.00) for DBP (*p* < 0.001) The mean CRP values decreased from 68.00 mg/dL (56.25–80.25) to 34.00 mg/dL (28.12–40.12) (*p* < 0.001), and the mean NT-proBNP decreased from 146.00pg/mL (122.50-170.50) to 136.00 pg/mL (112.50-160.50) (*p* = 0.005).

**Conclusion:**

Treatment with SGLT-2i in Romanian patients with T2DM has beneficial effects on modifiable cardiovascular risk factors.

## Introduction

Diabetes mellitus (DM) is a prevalent metabolic disorder affecting around 10% of the population in most countries [1]. The pathogenesis of diabetes is complex and understanding it and approaching a therapeutic approach that is as suitable as possible for each patient is extremely important.

Diabetes is a chronic pathology with a long evolution, so careful monitoring of patients and regular check-ups are essential parts of its management. The complications of diabetes are micro- and macrovascular, affecting cardiac function, renal function, the peripheral vascular system, and eye damage over time [2].

Globally, approximately 32.2% of patients with diabetes are affected by cardiovascular disease. Cardiovascular mortality is about 4.4 times higher in patients with diabetes who do not present classic cardiovascular risk factors (hypertension, smoking, dyslipidemia) compared to age-matched control subjects [3]. A study from Scotland and Verona demonstrated a 50% higher mortality risk than in patients without diabetes. It’s important to note that many patients with DM already have cardiovascular disease at the time of diagnosis [4].

Since mortality from cardiovascular causes through acute myocardial infarction and stroke represents a significant percentage both in patients with diabetes and in those who do not suffer from this condition, it is necessary to create a therapeutic scheme with the help of which to minimize as much as possible cardiovascular risk.

The risk factors associated with health and diseases can be behavioral (smoking, excessive alcohol consumption, diet, sedentary lifestyle) [5], physiological (overweight or obesity, hypertension, hypercholesterolemia, hyperglycemia) [6], demographic (age, gender, demographic subgroups) [7], environmental (pollution, occupational risks, social conditions) [8], genetics [9]. Together, these factors significantly increase the risk of chronic heart disease and other health problems. Aging populations and longer life expectancies have led to an increase in chronic (long-term) illnesses and disabilities, with higher treatment costs.

Cardiovascular risk factors include non-modifiable ones such as age, sex, genetic factors, ethnicity, and race. Modifiable ones include hypertension, smoking, diabetes, obesity, and dyslipidemia. Reducing the modifiable factors can improve mortality [10].

Because DM is an independent cardiovascular risk factor frequently associated with other risk factors such as hypertension, obesity, dyslipidemia, and even diabetic kidney disease, one of the main goals is to minimize this risk. This goal can be accomplished by modifying the risk factors, which can be achieved by making lifestyle changes and selecting the most appropriate therapy.

In multiple studies, sodium-glucose co-transporter 2 inhibitors (SGLT-2i) have shown cardio- and reno-protective effects. They promote weight loss, reduce blood pressure, reduce glycated hemoglobin and fasting blood sugar levels, improve kidney function by lowering albuminuria, and improve glomerular filtration rate [11]. SGLT-2i, a class of oral antidiabetics, has been shown to have increased efficacy in reducing modifiable cardiovascular risk factors and cardiovascular mortality (AMI, stroke) [12].

Multiple studies have demonstrated beneficial effects on the cardiovascular system. The present study aims to retrospectively analyze the impact of initiating treatment with SGLT-2i on modifiable cardiovascular risk factors and how it changed them. These aspects could contribute to making an appropriate therapeutic decision in patients with diabetes who present an increased cardiovascular risk.

## Patients and methods

### Study design and patients

This retrospective study includes 200 patients who followed a treatment with SGLT-2i, Dapagliflozin 10 mg (Forxiga (Dapagliflozin) 10 mg) /Qtern (Saxagliptin/Dapagliflozin 5/10 mg)), or Empagliflozin 10 mg (Jardiance 10 mg) for at least one year. The patients were chosen from the “Pius Brânzeu” Timisoara County Emergency Hospital based on the inclusion criteria, namely patients diagnosed with T2DM with SGLT-2i included in the treatment regimen.

The study was conducted following the guidelines of the Declaration of Helsinki, and the Ethics Committee of the “Pius Brinzeu” Emergency Hospital Timisoara approved the protocol. All the patients who participated in the study consented after being fully informed about the study’s details.

The inclusion criteria in the study were:

- patients with T2DM in treatment with different types of SGLT-2i and/or other DM medication.

The exclusion criteria from the study were:

- patients with T1DM;
- pregnant or lactating T2DM patients;
- the presence of a mental illness.

In this study, we tracked demographic data (sex, age, urban or rural origin), data obtained from the objective examination (blood pressure, weight, body mass index), and paraclinical data (fasting blood glucose, glycated hemoglobin, creatinine, rate of glomerular filtration, lipid profile).

The main modifiable risk factors monitored were body weight, blood pressure, glycemic balance (HbA1c, fasting glucose), lipid profile (HDLc, LDLc), and renal function (glomerular filtration rate, albuminuria).

### Anthropometric characteristics

The body mass index (BMI) was calculated by dividing a person’s weight in kilograms by the square of their height in meters. Body weight and height were precisely measured to the nearest 0.5 kg and 0.1 cm, respectively. Afterward, the patients were classified based on the obtained value into one of the following categories: normal weight (BMI between 18.5 and 14.9); overweight (BMI between 25 and 29.9); obesity degree I (BMI between 30 and 34.9); obesity degree II (BMI between 35 and 39.9); obesity grade III/morbid obesity (BMI over 40). Abdominal circumference (AC) was measured using a centimeter. Normal values were considered to be less than 80 cm for females and less than 94 cm for males [13].

AC was measured at the midpoint between the rib or costal margin and the iliac crest in the midaxillary line. For men, a waist circumference below 94 cm is considered low risk, between 94 and 102 cm is high risk, and over 102 cm is very high risk. For women, a waist circumference below 80 cm is considered low risk, 80–88 cm is high risk, and anything above 88 cm is considered very high risk [14].

Waist-stature ratio (WSR) represents waist circumference divided by height, > 0.5 for adults under 40 and > 0.6 for adults over 50 [14]. 

Blood pressure (BP) was measured in the left upper arm of seated subjects who had rested for 5 to 10 min. We used a conventional sphygmomanometer (Disytest, Germany) with an appropriate bladder size. Systolic BP (SBP) was determined as the average of the two measurements within 10 min. According to the 2019 European Society of Cardiology guidelines, depending on blood pressure values, patients are classified into the following categories: ideal blood pressure (< 120 systolic and < 80 diastolic), normal blood pressure (120–129 systolic and/or 80–85 diastolic), normal high blood pressure (130–139 and/or 85–89 diastolic), grade I hypertension (140–159 systolic and/or 90–99 diastolic), grade II hypertension (160–179 systolic and/or 100–109 diastolic), hypertension grade III (> 180 systolic and/or 110 diastolic) [15].

### Laboratory characteristics

The level of hemoglobin A1c (HbA1c) was measured using an immune turbidimetric assay that is standardized by the National Glycohemoglobin Standardization Program (NGSP) and compliant with the Diabetes Control and Complications Trial (DCCT). The assay was manufactured by Hoffman-La Roche Ltd. in Basel, Switzerland, and has an inter-measurement coefficient of variation of 1.64% as per the manufacturer’s specifications. The reference range was 4.8–6.4% [16].

The lipid profile was assessed using spectrophotometry (enzymatic-colorimetric), and the following reference values were set to total cholesterol < 200 mg/dL, triglycerides < 150 mg/dL, LDLc < 100 mg/dL, and HDLc > 50 mg/dL.

The plasma glucose was determined using an enzyme technique with glucose oxidase. We considered normal values for fasting glucose, between 70 and 110 mg%, and for postprandial glycemia, below 140 mg%. For the diagnosis of DM, we considered the criteria consisting of values equal to or greater than 126 mg% for fasting glucose and 200 mg for postprandial glycemia.

According to KDIGO classification, based on estimated glomerular filtration rate (eGFR), stages of Chronic Kidney Disease (CKD) are the following: stage one (I): if eGFR ≥ 90 ml/min/1.73 m^2^, stage two (II): if eGFR is between 60 and 89 ml/min/1.73 m^2^, stage three a (III-a): if eGFR is between 45 and 59 ml/min/1.73 m^2^, stage three b (III-b): if eGFR is between 30 and 44 ml/min/1.73 m^2^, stage four (IV): if eGFR is between 15 and 29 ml/ min/1.73 m^2^, and stage five (V): if the eGFR is < 15 ml/min/1.73 m^2^. A series of formulas based on age, sex, race, and serum creatinine (CKD-EPI, MDRD, etc.) are used to estimate GFR.12 In our study, the GFR estimation was made by applying the CKD-EPI formula. Serum creatinine was determined by Jaffe kinetics (colorimetric enzymatic method) as normal values at F < 1 mg% and B < 1.2 mg%. Urinary creatinine was determined by the spectrophotometric method (Colorimetric enzyme), assuming normal values of 600–2000 mg/day. Albuminuria was determined by the immunoturbidimetric method, considering the following interpretation: A1 < 30 mg/day: normal or slightly increased albuminuria, A2: 30–300 mg/day: moderate albuminuria, A3 > 300 mg/day: severe albuminuria. We performed the ratio of urinary albumin to urinary creatinine, with the following interpretation: A1 < 30 mg/g: normal, A2: 30–300 mg/g: moderate, A3 > 300 mg/g: severe. These data were obtained from the patient’s records, in which the analyses requested at the periodic check-up over several years were annexed. We followed the parameters change one year after the initiation of treatment with SGLT-2i and recorded their values before and at the end of the first year of treatment.

The urine albumin-to‐creatinine ratio (UACR) is most frequently applied to diagnose albuminuria. In spot urine specimens, normal level of UACR is below 30 mg/g. A 30 to 300 mg/g value in the spot urine is considered microalbuminuria, and more than 300 mg/g is considered macroalbuminuria [17]. 

C-reactive protein (CRP) was determined through the latex-immunoturbidimetry method. Normal values are under 0.5 mg/dl, and increased values show inflammation, bacterial infection, neoplasm, or severe trauma [18].

N-terminal pro b-type natriuretic peptide (NT-proBNP) was determined through the electrochemiluminescence method (ECLIA). It was found that the serum levels of BNP (and, respectively, of NT-proBNP) correlate very well with the severity of left ventricular dysfunction and with the NYHA (New York Heart Association) classes in which patients with HF (heart failure) are classified based on their clinical symptoms. The cut-off value of NT-proBNP is 125 pg/mL [19].

### Statistical analysis

The data were analyzed by calculating the mean and standard deviation for continuous variables Gaussian distribution and median (inter-quartile range) for continuous variables without non-Gaussian distribution. In addition, the frequency (percentage) was computed for nominal variables. Patient groups were compared, considering the type of variables. Continuous variables were compared using the Student’s *t-*test for Gaussian populations and Mann–Whitney *U* test for non-Gaussian populations, while nominal variables were compared using the chi-square test or Fisher’s exact test. The condition of normality of continuous variable distribution was tested using the Shapiro–Wilk test, while the equality of variances was tested using Levene’s test.

The statistical analysis was conducted using MedCalc® Statistical Software version 20.106 (MedCalc Software Ltd., Ostend, Belgium). The graphical representations were generated using GraphPad Prism version 10.1.2 software. The significance threshold was considered *p*-value < 0.05.

## Results

**Fig. 1:**
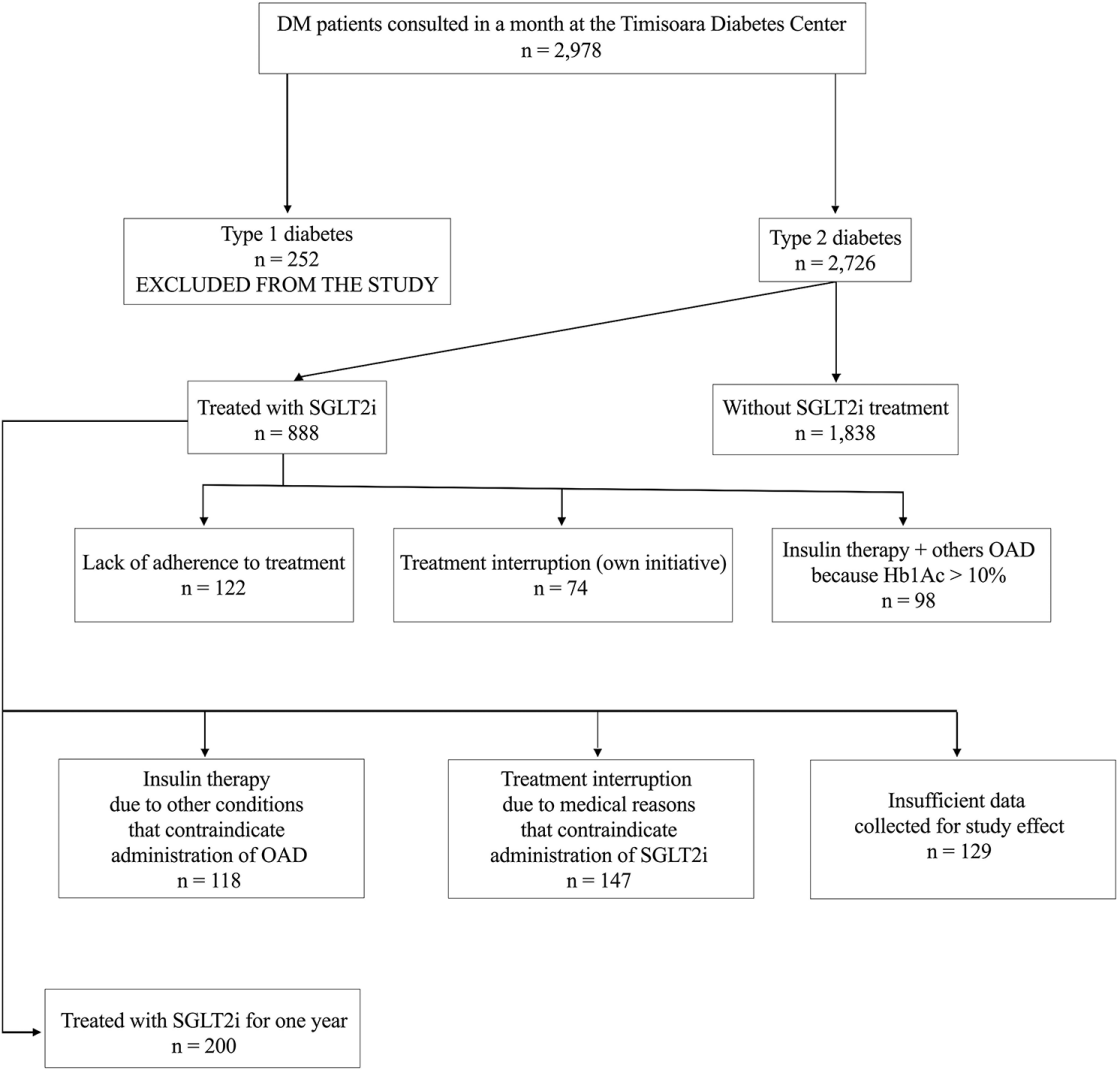
Flow diagram of the patients enrolled in the study. *Abbreviations*: T1DM - Type 1 diabetes mellitus, T2DM - type 2 diabetes mellitus, SGLT-2i - sodium glucose 2 co-transporter inhibitors

The study group included 200 patients with T2DM (Figs. 1), 44% male and 56% female (*p* = 0.089, X^2^ = 2.88). Patients aged between 40 and 95 years were included in the present study, with a mean age of 63.95 and a standard deviation (*SD* of ± 10.29). Depending on the environment of origin, 61% came from an urban environment and 39% from a rural environment (*p* = 0.0018, X^2^ = 9.68) (Table 1).

**Table 1:** Characteristics of the studied sample

Parameters	*N* = 200
Age (years)^a^	63.9 ± 10.29
**Gender** ^b^	
Feminine	56 (56%)
Masculin	44 (44%)
**Environment** ^b^	
Urban	61 (61%)
Rural	39 (39%)

^a^Continuous variables (with distribution) are indicated by their mean ± SD.

^b^Categorical variables are presented by percentage (absolute frequency) in the sample

We obtained beneficial effects of SGLT-2i on modifiable cardiovascular risk factors, as shown in Table 2.

**Table 2:** Effects of SGLT-2i on modifiable risk factors before and after one year of treatment

Parameters^a^	Before	After	*p*-value^b^
**Effects on glycemic balance**			
Fasting blood glucose (mg%)	180.00 (154.50–207.00)	130.00 (117.50–150.00)	< 0.001
HbA1c (%)	8.40 (7.98–9.15)	7.30 (6.90–7.95)	< 0.001
**Effects on body weight**			
Weight (kg)	90.50 (82.00-106.50)	89.00 (77.50–100.00)	0.018
BMI (kg/m^2^)	32.87 (29.24–36.45)	31.00 (27.74–34.71)	< 0.001
AC (cm)	107.05 ± 16.39	102.50 ± 15.11	0.042
WSR	1.67 (1.60–1.74)	2.79 (2.56–3.01)	0.057
**Effects on the cardiovascular system (blood pressure and cardiac failure)**			
SBP (mmHg)	140.00 (130.00-160.00)	120.00 (115.50–130.00)	< 0.001
DBP (mmHg)	80.00 (72.00–90.00)	72.00 (70.00–78.00)	< 0.001
NT-proBNP (pg/mL)	146.00 (122.50-170.50)	136.00 (112.50-160.50)	0.005
**Effects on renal function**			
GFR (ml/min/1.73 m^2^)	75.00 (60.00-88.50)	89.00 (70.00–92.00)	< 0.001
Albuminuria (mg/day)	179.00 (163.00-251.25)	169.00 (12.00-184.00)	0.002
UACR (mg/g)	23.98 (19.76–36.85)	19.39 (1.30-24.29)	< 0.001
**Effects on lipid metabolism**			
HDLc (mg%)	45.00 (37.00–52.00)	50.00 (43.00-56.50)	0.039
LDLc (mg%)	88.50 (69.50-103.50)	91.50 (70.00-114.85)	0.640
**Effects on inflammation**			
CRP (mg/dL)	68.00 (56.25–80.25)	34.00 (28.12–40.12)	< 0.001

*Abbreviations* HbA1c: hemoglobin A1c; BMI: body mass index; AC: abdominal circumference; WSR: waist-stature ratio; SBP: systolic blood pressure; DBP: diastolic blood pressure; NT-proBNP: N-terminal pro b-type natriuretic peptide; GFR: glomerular filtration rate; UACR: urine albumin-to‐creatinine ratio; CRP: C-reactive protein.^a^Continuous variables with Gaussian distribution are indicated as mean (± st.dev.), continuous variables with non-Gaussian distribution are indicated as median (interquartile range) and categorical variables, as percentage (absolute frequency) in the sample.^b^p-value was computed by Mann–Whitney U test for continuous variables (with non-Gaussian distribution) and Pearson’s chi-squared (or Fisher’s exact) test for nominal variables

**Fig. 2:**
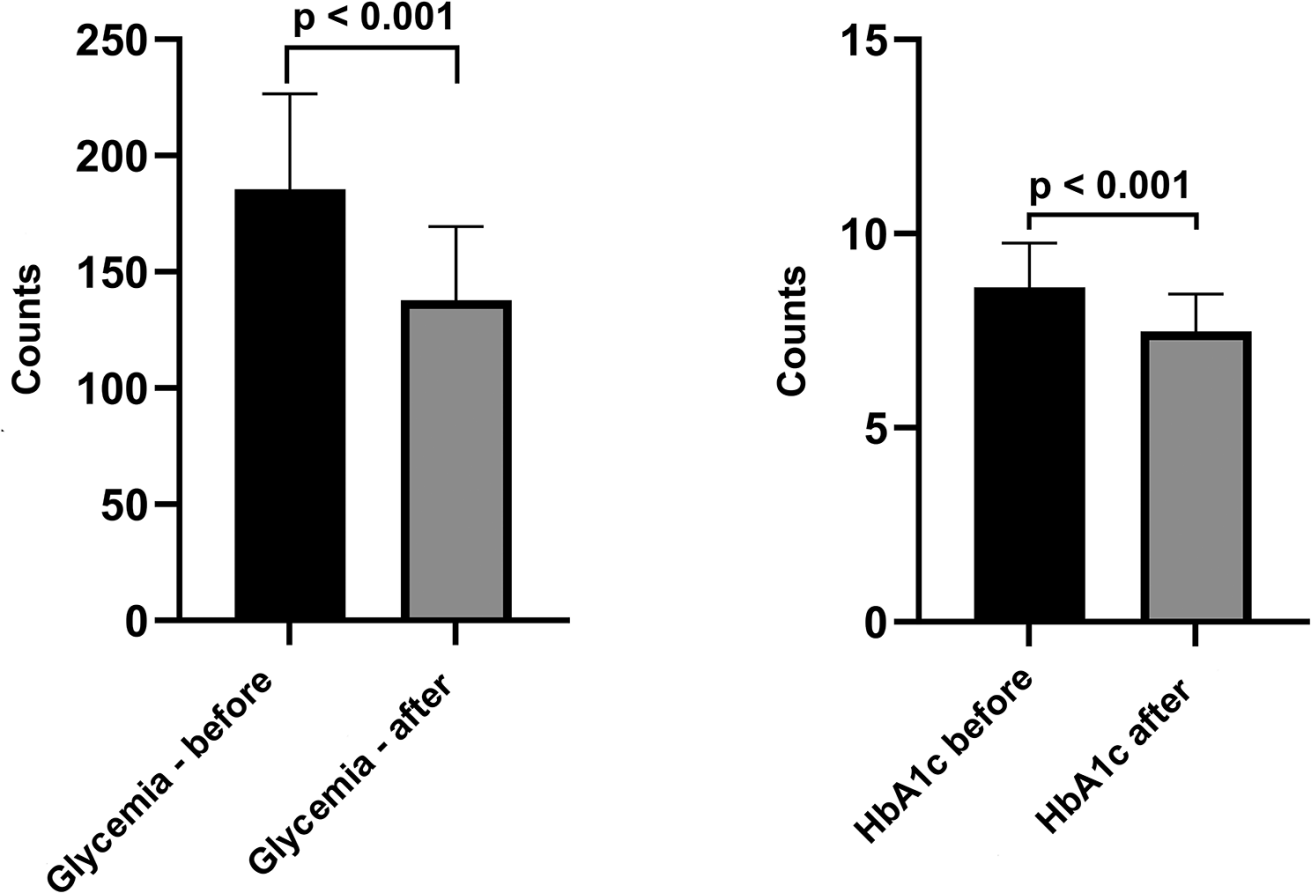
Effects of SGLT-2i on glycemic balance. **(a)** Comparison of mean fasting blood glucose values before and after the treatment. **(b)** Changes in mean HbA1c before and after treatment

The effect of SGLT-2i on glycemic balance was studied by the impact on fasting blood glucose and HbA1c. Diabetes mellitus, defined as a persistent hyperglycemic state, is an independent cardiovascular risk factor; thus, in patients with diabetes, it is essential to reduce fasting glucose values. In this study, we followed the fasting blood glucose values before and one year after the initiation of treatment with SGLT-2i. Before the initiation of inhibitor therapy, we had a median value of FBG of 180.00 mg% (154.50–207.00), and after one year of treatment, 130.00 mg% (117.50–150.00) (*p* < 0.001) (Fig. 2a).

HbA1c is a useful measurement in evaluating and monitoring patients with DM. This shows us an average of the glycemic values of the last three months, independent of diet or blood glucose fluctuations. Thus, the monitoring of the patient’s adherence to treatment or the effectiveness of treatment can be achieved. The target glycated hemoglobin in patients with DM is 7%, and its normal values in patients without diabetes are below 6.4%. We analyzed HbA1c values before and after the treatment, and the results obtained are as follows: the mean of HbA1c values decreased from 8.40% (7.98–9.15) to 7.30% (6.90–7.95) (*p* < 0.001) (Fig. 2b).

**Fig. 3:**
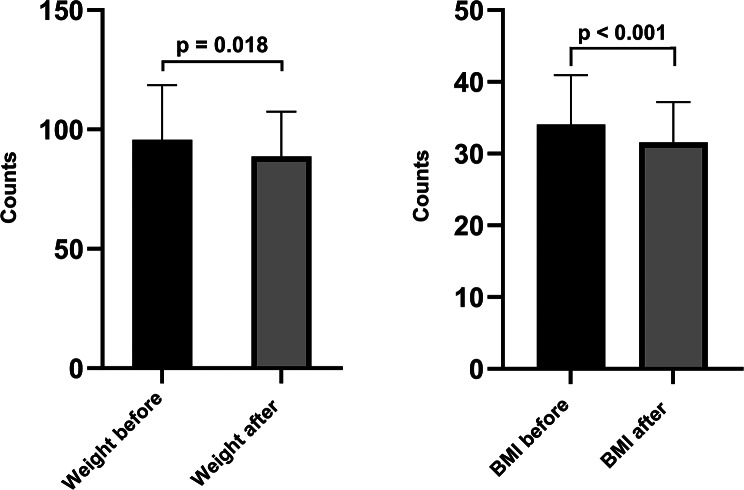
Impact of SGLT-2i on body weight (**a**) weight and (**b**) BMI.Abbreviations: BMI: body mass index

SGLT-2i has a beneficial effect on body weight, reducing its values. The impact of SGLT-2i on body weight was appreciated in terms of weight, BMI, AC, and WSR. After one year of treatment with SGLT-2i, we obtained a significant decrease in all tree parameters as follows: weight from 90.50 kg (82.00-106.50) to 89.00 kg (77.50–100.00) (*p* = 0.018), BMI from 32.87 kg/m^2^ (29.24–36.45) to 31.00 kg/m^2^ (27.74–34.71) (*p* < 0.001) and AC from 107.05 cm (± 16.39) to 102.50 cm (± 15.11) (*p* = 0.042). (Fig. 3). We did not obtain significant results when comparing AC and WSR by gender.

The beneficial effect of SGLT-2i, namely the decrease in its values, is well known. It is recommended for patients with T2DM who have associated hypertension. Thus, we made two graphs comparing blood pressure values ​​before and one year after treatment with Dapaglifozin or Empaglifozin.

**Fig. 4:**
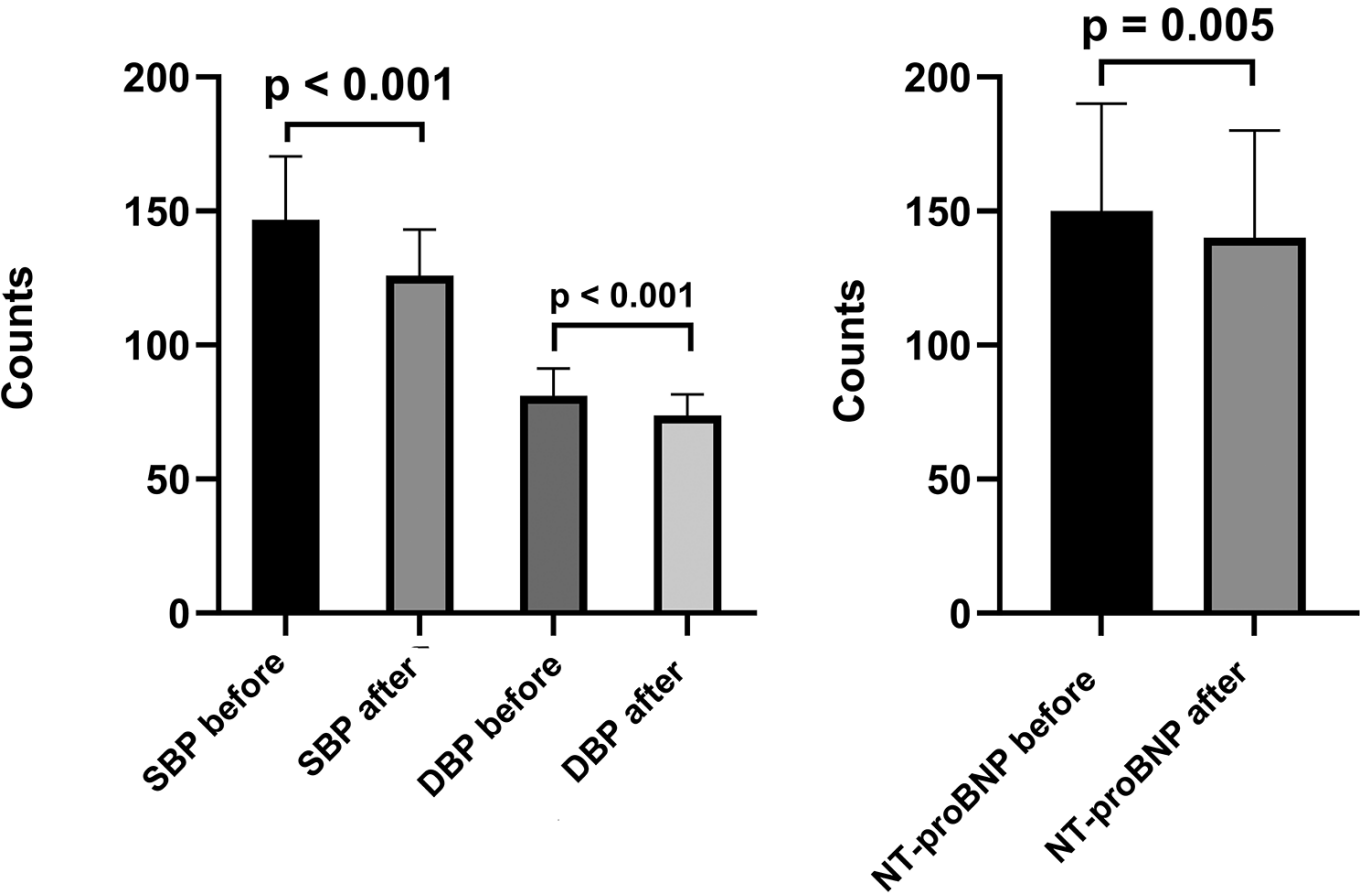
Impact of SGLT-2i on the cardiovascular system.*Abbreviations*: SBP: systolic blood pressure, DBP: diastolic blood pressure, NT-proBNP: N-terminal pro b-type natriuretic peptide

Initially, the median value for SBP was 140.00mmHg (130.00-160.00), and for DBP was 80.00 mmHg (72.00–90.00). At one year after treatment, the median value was 120.00 mmHg (115.50–130.00) for SBP (*p* < 0.001) and 72.00 mmHg (70.00–78.00) for DBP (*p* < 0.001) (Fig. 4).

Depending on the GFR values, patients are placed in a specific category, with the help of which, along with the categories of albuminuria, the risk of developing CKD can be quantified: stage G1 (GFR ≥ 90 ml/min/1.73m2), stage G2 (GFR 60–89 ml/min/1.73m2), stage G3a (GFR 45–59 ml/min/1.73m2), stage G3b (GFR 30–44 ml/min/1.73m2), stage G4 (GFR 15–29 ml/min/1.73m2), stage G5 (GFR < 15 ml/min/1.73m2). The stages of albuminuria are represented by A1 (< 30 mg/g), A2 (30–300 mg/g) and A3 (> 300 mg/g).

**Fig. 5:**
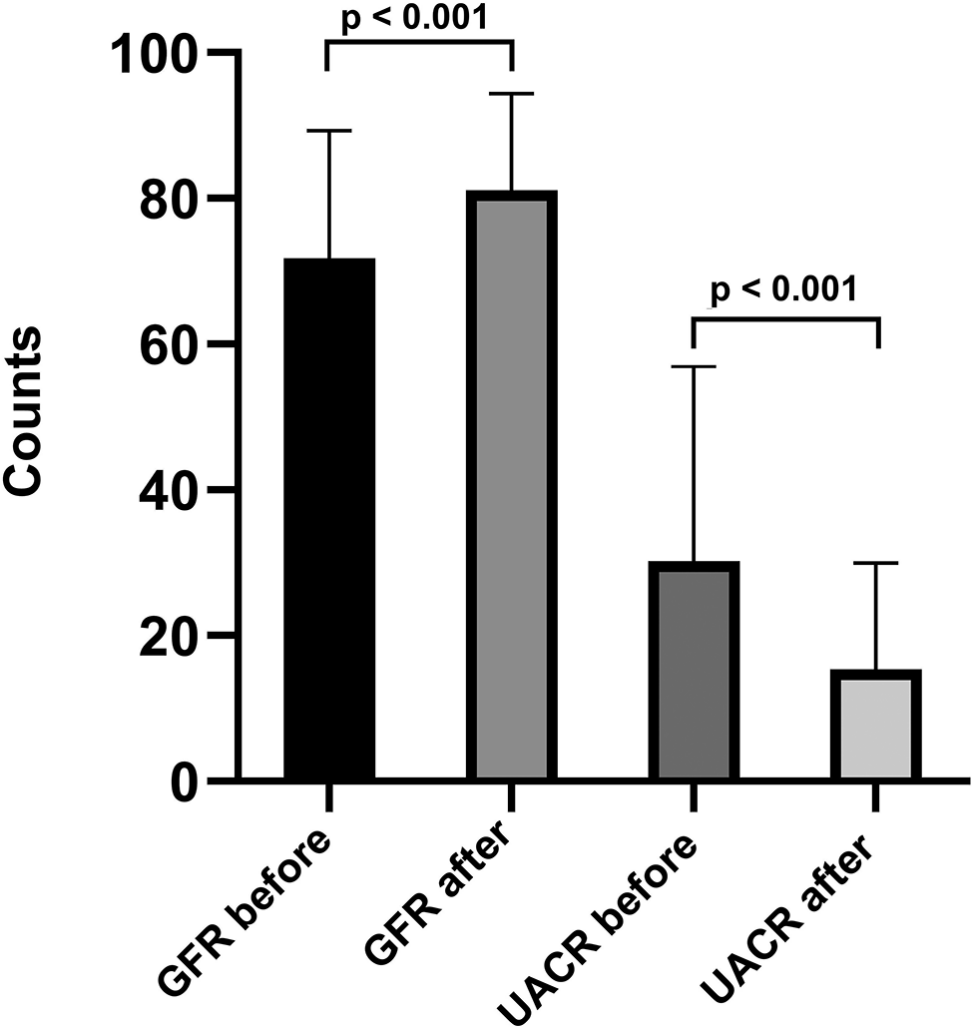
Effects of SGLT-2i on renal function. GFR changes before and after treatment, and changes in UACR before and after the treatment. *Abbreviations* GFR: glomerular filtration rate, UACR: urine albumin-to‐creatinine ratio

We analyzed the GFR, albuminuria, and UACR values before and after treatment following their changes. The GFR increased from 75.00 (60.00-88.50) to 89.00 (70.00–92.00) (*p* < 0.001), albuminuria decreased from 179.00 (163.00-252.00) to 169.00 (12.00-184.00) (*p* = 0.002), and UACR decreased from 23.98 mg/g (19.76–36.85) to 19.39 mg/g (1.30-24.29) (*p* < 0.001) (Fig. 5).

We analyzed the HDL and LDL cholesterol values before and one year after initiation of treatment with SGLT-2i. The results obtained were as follows: the median HDL values increased from 45.00 mg% (37.00–52.00) to 50.00 mg% (43.00-56.50) (*p* = 0.039), while median LDL cholesterol increased from 88.50 mg% (69.50-103.50) to 91.50 mg% (70.00-114.85) (*p* = 0.640).

Also, beneficial effects were obtained on CRP, a marker of inflammation, and NT-proBNP, a marker of cardiac insufficiency. Both markers presented a statistically significant decrease after one year of treatment with SGLT-2i. The median CRP values decreased from 68.00 mg/dL (56.25–80.25) to 34.00 mg/dL (28.12–40.12) (*p* < 0.001), and the median NT-proBNP decreased from 146.00pg/mL (122.50-170.50) to 136.00pg/mL (112.50-160.50) (*p* = 0.005).

## Discussion

In the group of patients selected in the present study, the maximum prevalence of diabetes was found in the age group of 60–69 years with a percentage of 35%. This incidence was similar in the specialized literature, with results close to those obtained in this study. The PREVADIAB study (First diabetes prevalence study in Portugal) from Portugal showed a maximum incidence in the age group between 60 and 79 years with a percentage of 26.3% [20].

Considering the prevalence of diabetes according to the sex of the patients and the inclusion in the therapeutic scheme of SGLT-2i, it turned out to be more frequent in the female sex. The same results were also obtained in other studies, such as in the CANVAS (Canagliflozin Cardiovascular Assessment Study) study, where a higher frequency of diabetes was found in females (45%) compared to males (31%) [21].

According to the area of ​​origin, the study results show a difference between the prevalence of diabetes in urban areas versus rural areas, with diabetes being more common in urban areas. Like this result, we have other studies in which it has been demonstrated that diabetes is more common in the urban environment compared to the rural one [22].

Following the EMPA-REG OUTCOME study, cardioprotective aspects were proven by introducing an SGLT-2i into the therapeutic scheme of patients with T2DM. Through the effects of this class of oral antidiabetics, arterial stiffness is reduced, and natriuresis and glycosuria are promoted, thus decreasing the values of blood pressure without increasing heart rate and promoting weight loss [23].

In the literature, studies in both hypertensive and normotensive patients with T2DM that demonstrate a 4–10 mmHg reduction of systolic blood pressure [24, 25] are also cited. The present study also found a decrease in blood pressure, where the percentage of patients with optimal and normal blood pressure increased.

Glycosuria promoted by treatment with SGLT-2i contributes to weight loss. We demonstrated the beneficial effects of reducing body weight (a modifiable cardiovascular risk factor) in patients with diabetes. BMI was significantly reduced in study patients. The percentage of normal-weight and overweight patients increased due to the decrease in the percentage of those classified as obese. Multiple studies support this information, one of which also explains the mechanisms by which SGLT-2i reduces body mass index [26]. Studies have shown that SGLT-2i treatment alone or combined with other GLDs can lead to weight loss in Western populations. Specifically, the weighted mean difference compared to placebo is -1.74 kg with a 95% confidence interval of -2.03 to -1.45, while the weighted mean difference compared to other GLDs is -1.80 kg with a 95% CI of -3.50 to -0.11 kg [27]. Patients with T2DM from Asia had similar results. SGLT-2i treatment led to significant weight loss, ranging from − 0.5 to -3.9 kg over a study duration of 8 to 104 weeks [28].

The glycemic profile improved significantly in the patients followed in this study, resulting in a lower HbA1c value over one year. Various studies have supported this beneficial effect of SGLT-2i by demonstrating a reduction in glycated hemoglobin from a mean of 8.9 ± 1.7% to 8 ± 1.5% over a 6-month period [29]. In a Japanese study, among 165 patients, SGLT2 inhibitor treatment decreased HbA1c from 8.2 to 7.6% after 12 weeks [30].

In addition to the cardioprotective effects, several studies noted favorable effects on renal function. A reduction in the value of albuminuria (a marker of kidney damage) in patients with impaired renal function and a reduction in its worsening over time is noted. A study shows that 32.6% of patients treated with empagliflozin went from the stage of macroalbuminuria to microalbuminuria or from micro- to non-albuminuria compared to the placebo group, of which only 8.6% of changes were found. Similarly, in the study carried out in the Timisoara County Emergency Hospital, from a percentage of 56% of patients classified as having normoalbuminuric, an increase to 71% was noticed [31].

The reno-protective effects of SGLT-2i in people with T2DM have been evaluated in five major cardio-vascular (CV) outcomes trials (CVOTs): EMPA-REG OUTCOME (empagliflozin) [32], CANVAS program, which comprised two randomized, double-blind, placebo-controlled phase 3 trials: CANVAS and CANVAS-R (canagliflozin) [33], DECLARE-TIMI 58 (dapagliflozin) [34], and VERTIS CV (ertugliflozin) [35]. Each trial evaluated a composite of renal events as outcome measures, typically secondary endpoints, including “hard” endpoints such as ESKD or renal death. In addition, several studies have used UACR, eGFR, and serum uric acid levels as markers of overall renal risk in patients with varying levels of renal risk. These CV outcomes studies suggest that SGLT-2i can prevent the development of CKD and delay its progression in individuals with T2DM, regardless of their level of renal risk. The CREDENCE trial focused on individuals with CKD and T2D [36], while the DAPA-CKD trial included people with CKD with or without T2D [37]. The main objective of these trials was to examine the impact of SGLT-2i on renal outcomes. The results of both trials showed that SGLT-2i can significantly lower the risk of worsening CKD.

Renoprotective effects are also found in the DAPA-HF study, which reported an increase in the glomerular filtration rate with a reduction in the renal composite established at the beginning of the study. In the study presented above, a decrease in BCR stage G4 from 3 to 15%, G3a stage from 15 to 12%, and G2 from 55 to 44% was achieved in the DAPA-HF study [38].

One of the adverse effects of SGLT-2i is changes in the lipid profile, with a slight increase in HDL and LDL cholesterol but without a significant increase in the LDL/HDL cholesterol ratio [39]. This study also noted an increase in HDL and LDL cholesterol values. We also wanted to verify the hypothesis of an insignificant rise in the LDL/HDL cholesterol ratio, and we obtained a decrease of 0.12%.

Several studies have shown that SGLT-2i can reduce the serum levels of total cholesterol and triglyceride [40, 41]. However, whether SGLT-2i treatment can reduce the serum levels of HDL and LDL cholesterol remains controversial. Cha et al. conducted an observational study to compare the effects of SGLT-2i and dipeptidyl-peptidase IV inhibitors in patients with DM treated with metformin or sulfonylurea. The study found that after using SGLT-2i as add-on therapy, there was a significant increase in LDL cholesterol levels compared to the effects of dipeptidyl-peptidase IV inhibitors [42]. In a randomized controlled trial conducted on diabetic patients, Schernthaner et al. found that canagliflozin treatment significantly increased serum LDL cholesterol levels compared to sitagliptin [43]. However, studies have yet to be conducted to investigate the effect of SGLT-2i on high-risk lipid profiles, mainly oxidative LDL. The inconsistent results among studies investigating the impact of SGLT-2i on the lipid profile, including those reported herein, may be due to differences in the characteristics of study participants rather than the different drugs used [44].

In our study, we follow the effect of SGLT-2i on PCR with positive results. In the literature are studies about the impact of SGLT-2i on inflammatory markers (IL-6, CRP, TNF-α, and MCP-1) in animal models, with positive effects [45].

Our study also obtained positive effects of SGLT-2i on NT-proBNP. The effect of SGLT-2i on NT-proBNP is controversial in the literature. Some studies have reported favorable outcomes [46, 47], while others have shown harmful or no effects [48, 49]. In the EMPEROR-Reduced Trial, treatment with empagliflozin lowered NT-proBNP concentrations [50].

Since studies have shown a reduction in the risk of hospitalization for HF in type 2 DM patients treated with SGLT2i, they should be included in the first stage of HF treatment [51].

A series of experimental studies have proven that SGLT2i have antioxidant and anti-inflammatory effects, at the macro- and microvascular level, including at the level of the coronary microvascular endothelium [52]. Thus, the beneficial effects of SGLT2i on the endothelial function whose alteration determines the appearance of atherosclerosis and cardiovascular damage over time have been proven [53].

A limitation of this study is the small sample size; however, all the patients with T2DM were volunteers, and their computer literacy and internet competence conditioned their participation in the study. The strengths of our study include the novelty of the study design, being the first study to study the impact of SGLT-2i on modifiable cardiovascular risk factors in Romanian patients with T2DM.

## Conclusions

In conclusion, treatment with SGLT-2i in patients with T2DM has beneficial effects on modifiable cardiovascular risk factors. The prevalence of T2DM is increasing, and individuals with this condition have a higher risk of developing CVD. To manage patients who have established CVD or are at high risk of CV disease, SGLT-2i is a novel and important therapeutic option for clinicians. Considering their consistent benefits in preventing heart failure hospitalization and cardiovascular disease, SGLT-2i should be included early in the treatment plan for patients with multiple risk factors or established CVD. The ongoing studies on the mechanisms and outcomes of SGLT-2i in patients with HF and CKD will help determine its cardiorenal protective role beyond glucose lowering.

## Data Availability

The used datasets and/or analysed during the current study are available with the corresponding author upon reasonable request.

## Abbreviations

SGLT-2i: Sodium-Glucose 2 Co-Transporter Inhibitors
T2DM: Type 2 Diabetes Mellitus
BMI: Body Mass Index
FBG: Fasting Blood Glucose
HbA1c: Glycated Hemoglobin
Ac: Abdominal Circumference
UACR: Urine Albumin-To‐Creatinine Ratio
SBP: Systolic Blood Pressure
DBP: Diastolic Blood Pressure
CRP: C-Reactive Protein
NT-proBNP: N-Terminal Pro B-Type Natriuretic Peptide
HF: Heart Failure
CKD: Chronic Kidney Disease
eGFR: Estimated Glomerular Filtration Rate
CV: Cardio-Vascular
CVD: Cardio-Vascular Disease
